# Changes of Structural Brain Network Following Repetitive Transcranial Magnetic Stimulation in Children With Bilateral Spastic Cerebral Palsy: A Diffusion Tensor Imaging Study

**DOI:** 10.3389/fped.2020.617548

**Published:** 2021-01-15

**Authors:** Wenxin Zhang, Shang Zhang, Min Zhu, Jian Tang, Xiaoke Zhao, Ying Wang, Yuting Liu, Ling Zhang, Hong Xu

**Affiliations:** ^1^Department of Rehabilitation, Children's Hospital of Nanjing Medical University, Nanjing, China; ^2^Department of Radiology, Children's Hospital of Nanjing Medical University, Nanjing, China

**Keywords:** cerebral palsy, diffusion tensor imaging, structural brain network, graph theory, repetitive transcranial magnetic stimulation

## Abstract

**Introduction:** Bilateral spastic cerebral palsy (BSCP) is the most common subtype of cerebral palsy (CP), which is characterized by various motor and cognitive impairments, as well as emotional instability. However, the neural basis of these problems and how repetitive transcranial magnetic stimulation (rTMS) can make potential impacts on the disrupted structural brain network in BSCP remain unclear. This study was aimed to explore the topological characteristics of the structural brain network in BSCP following the treatment of rTMS.

**Methods:** Fourteen children with BSCP underwent 4 weeks of TMS and 15 matched healthy children (HC) were enrolled. Diffusion tensor imaging (DTI) data were acquired from children with bilateral spastic cerebral palsy before treatment (CP1), children with bilateral spastic cerebral palsy following treatment (CP2) and HC. The graph theory analysis was applied to construct the structural brain network. Then nodal clustering coefficient (*C*_*i*_) and shortest path length (*L*_*i*_) were measured and compared among groups.

**Results:** Brain regions with significant group differences in *C*_*i*_ were located in the left precental gyrus, middle frontal gyrus, calcarine fissure, cuneus, lingual gyrus, postcentral gyrus, inferior parietal gyri, angular gyrus, precuneus, paracentral lobule and the right inferior frontal gyrus (triangular part), insula, posterior cingulate gyrus, precuneus, paracentral lobule, pallidum. In addition, significant differences were detected in the *L*_*i*_ of the left precental gyrus, lingual gyrus, superior occipital gyrus, middle occipital gyrus, superior parietal gyrus, precuneus and the right median cingulate gyrus, posterior cingulate gyrus, hippocampus, putamen, thalamus. *Post hoc t*-test revealed that the CP2 group exhibited increased *C*_*i*_ in the right inferior frontal gyrus, pallidum and decreased *L*_*i*_ in the right putamen, thalamus when compared with the CP1 group.

**Conclusion:** Significant differences of node-level metrics were found in various brain regions of BSCP, which indicated a disruption in structural brain connectivity in BSCP. The alterations of the structural brain network provided a basis for understanding of the pathophysiological mechanisms of motor and cognitive impairments in BSCP. Moreover, the right inferior frontal gyrus, putamen, thalamus could potentially be biomarkers for predicting the efficacy of TMS.

## Introduction

Cerebral palsy (CP) is considered as an early childhood-onset neurodevelopmental disorder, which is characterized by motor and postural dysfunction often accompanied by cognitive impairments (1, 2). Bilateral spastic cerebral palsy (BSCP) is one of the most common CP subtype, which can also lead to cognitive disabilities and learning disabilities (3, 4). Repetitive transcranial magnetic stimulation (rTMS) is a non-invasive neuromodulation technique, which has been widely used for rehabilitation of CP (5, 6). In addition, the emerging magnetic resonance imaging (MRI) technique, especially diffusion tensor imaging (DTI), provides a non-invasive tool to explore white matter changes of CP and has recently advance our current understanding of the pathogenesis of CP (7–9). Therefore, there is a need to explore the effect of rTMS in the treatment of CP and identify changes of the brain networks that were modulated by the rTMS intervention.

DTI is a sensitive method to detect the microstructure of white matter, which allows the reconstruction of neuronal pathways (10–12). Previous DTI studies identified moderate and severe white matter abnormalities in the brain of CP, which could predict the neurocognitive and behavioral impairments of patients (13, 14). Children with spastic CP were found to be associated with the corticospinal tract lesions, which might lead to the motor impairments and help the early and highly accuracy diagnosis of spastic CP (14–16). In addition, the abnormal structure of basal ganglia and thalamus were found in CP children by the method of MRI, which were considered to be related to the motor and postural dysfunction of CP (17, 18). However, the abnormalities of a single white matter tract or an independent brain region could not able to reflect the pathophysiological mechanisms of motor and cognitive dysfunction in CP. Widespread abnormalities were found in the cerebral cortex and subcortical structures of CP children.

Graph theory provides a powerful mathematical framework and some metrics, which can describe the topological features of the brain networks. The brain network consisted of nodes (brain areas) and edges (structural connections between brain areas) can reflected the connectivity of all regions in the brain. Previous DTI-based brain network study showed that CP children had aberrant nodal parameters in the sensorimotor cortex (19). Distributed network-level structural disruptions and widespread changed topological characteristics of local brain regions might be associated with the pathogenesis of neurosensory and cognitive impairments of CP, such as the posterior-anterior neural network (20, 21). Moreover, abnormal nodal parameters were found in the prefrontal cortex, motor areas, cingulate gyrus and occipital cortex of CP, which might contribute to the motor dysfunction of patients, as well as cognitive impairments (19). Moreover, substantial functional reorganization of the motor cortex and corticospinal projections were found in CP children after the treatment of rTMS, which demonstrated that it was a possible treatment for neurodevelopmental disorders (6). However, little is known about how the rTMS modulates the topological characteristics of the structural brain network in BSCP children.

The present study was aimed to perform a DTI based investigation of topological characteristics of the structural brain network in BSCP following the treatment of rTMS. Based on the findings of previous studies, we hypothesized that BSCP children might had improved motor function, as well as changes of structural brain connectivity after the treatment of rTMS.

## Materials and Methods

### Participants

A total of 14 children with BSCP were recruited from the department of rehabilitation in Children's Hospital of Nanjing Medical University. The clinical features and MRI scanning of participants were used for the diagnosis of BSCP through standardized assessment by two neurologists. Fifteen healthy children (HC) age- and sex-matched without neurological and psychiatric disorders were recruited. In addition, the Gross Motor Function Classification System (GMFCS) (22) and Gesell Developmental Schedules (Gesell) (23) were conducted to assess the level of motor and cognitive function of all participants including patients before and following the treatment of rTMS.

The inclusion criteria were as follows: (1) diagnosis of BSCP according to strict criteria; (2) right-handed; (3) age≤24 months; (4) no history of trauma or brain operation; (5) no abnormal neuroimaging findings by conventional MRI. The exclusion criteria were as follows: (1) congenital developmental malformations; (2) genetic metabolic diseases; (3) history of nervous system infection; (4) psychiatric disorders; (5) disorders hearing abnormalities; (6) severe visual difficulties.

This study was approved by the Ethics Committee of the Children's Hospital of Nanjing Medical University. The consents were obtained from a parent or guardian on behalf of the participants.

### Repetitive Transcranial Magnetic Stimulation

All children received 10 rTMS sessions daily for 2 weeks with a 70-mm figure-of-eight coil magnetic stimulator (YIRUIDE, Wuhan, China) (24). The human cerebral cortex was a complex system with tightly interconnected excitatory and inhibitory neuronal networks. High-frequency (HF) (>5-Hz) rTMS increased cortical excitability, whereas low-frequency (LF) (<1-Hz) rTMS decreased cortical excitability. In addition, high frequency stimulation could enhance cortical excitability, which resulted in improvement in motor function. Low frequency stimulation had an inhibitory effect on the brain function, which decreased motor function. Therefore, the stimulation targeted site was located at left dorsolateral prefrontal cortex (lDLPFC), which was considered to play an important role in the regulation of motor and cognitive function (25, 26). The parameters of rTMS treatment were set based on previous study (27), which demonstrated that the treatment of 10-Hz rTMS could improve the motor and cognitive function of patients. The parameters of rTMS were as follows: 10 Hz, 3 s stimulation, 27 s interval, 110% intensity of resting motor threshold (rMT), 900 pulses per session, a total of 15 min/session.

### MRI Data Acquisition

The MRI data of the CP1, CP2 and HC groups were obtained using a 3.0 Tesla Philips MRI scanner at the department of radiology of Children's Hospital of Nanjing Medical University, Nanjing, China. The scan parameters of 3D T1-weighted imaging were as follows: repetition time (TR) = 7.9 ms, echo time (TE) = 3.5 ms, slice thickness = 1 mm, field of view (FOV) = 200 × 200 mm^2^, matrix size = 200 × 200, flip angle (FA) = 7.8°. The scan parameters of DTI imaging were as follows: TE = 96 ms, TR = 4,596 ms, slice thickness = 2 mm, FOV = 200 × 200 mm^2^, matrix size = 100 × 98, FA = 90°.

### MRI Data Preprocessing

The MRI data were preprocessed with the diffusion toolbox of Functional MRI of the Brain (FMRIB) software Library (FSL) (28). Firstly, eddy current and motion artifact correction of the DTI data were performed. Secondly, estimation of the diffusion tensor was performed. Finally, the fractional anisotropy (FA) of each voxel was calculated and then diffusion tensor tractography was performed. Diffusion tensor tractography was implemented using the Diffusion toolkit (http://trackvis.org/dtk/) by the fiber assignment by continuous tracking (FACT) algorithm. The tracts were computed by seeding each voxel with an FA > 0.2. The tractography was terminated if it reached a voxel with an FA < 0.2 or turned an angle >50°.

### Brain Network Construction

To define the nodes of the structural brain network, the whole brain were segmented by the automated anatomical labeling template (AAL) template (29). Then 90 cortical and subcortical regions (45 for each hemisphere) were obtained, which represented the nodes of the brain network. To define the edges between nodes of the brain network, a threshold was selected for the fiber bundles. Two regions were considered structurally connected if there were at least 3 fibers and two endpoints were located in these two regions. Using this procedure, the edges of the brain network were also obtained.

### Nodal Characteristics Analysis

Graph theory was used to quantify the nodal characteristics of the structural brain network, which included the nodal clustering coefficient and shortest path length (30). The clustering coefficient of the node (*C*_*i*_) is defined as the likelihood that the node's neighborhoods are connected with each other, which is often used to investigate the segregation of the node in the network. The shortest path length between nodes is defined as the length of the path with the shortest length between nodes. The shortest path length of the node (*L*_*i*_) is defined as the mean of shortest path length between the node *i* and all other nodes, which is often used to investigate the integration of the node in the network. Therefore, *C*_*i*_indicates the extent of the local interconnectivity of the node *i* and quantifies the local efficiency of information transfer of the node *i* (the local efficiency) while *L*_*i*_ quantifies the ability of the node *i* to propagate information in parallel (the global efficiency).

### Statistical Analysis

Two sample *t*-test was applied to compare the demographic and clinical data between groups. One-way analysis of variance (ANOVA) was performed to compare the values of *C*_*i*_ and *L*_*i*_ among three groups. Then *post hoc t*-test was applied to identify the differences of *C*_*i*_ and *L*_*i*_ between groups. A false discovery rate (FDR) procedure was applied to address the problem of multiple comparisons at a *q* value of 0.05. The significant level was set at *P* < 0.05 for all statistical tests.

## Results

### Demographic and Clinical Characteristics

The clinical information of the CP1, CP2 and HC groups were presented in Table 1. There were no significant differences in gender (*P* = 0.72) and age (*P* = 0.78) between groups. The CP1 group had lower total scores of Gesell when compared with the groups of CP1 and HC (*P* < 0.00) groups.

**Table 1:** Demographic and clinical characteristics.

**Characteristics**	**CP1 (*N* = 14)**	**CP2 (*N* = 14)**	**HC (*N* = 16)**	***P***
Age (months, mean ± SD)	16.64 ± 4.70	16.64 ± 4.70	15.47 ± 4.26	0.72
Gender (N)	9 M/5 F	9 M/5 F	8 M/7 F	0.78
Gesell (mean ± SD)	59.50 ± 8.11	67.79 ± 9.00	96.20 ± 5.91	0.00
GMFCS (N)	II: 4; III: 7; IV: 3	II: 9; III: 5	–	–

*CP1, children with bilateral spastic cerebral palsy before treatment; CP2, children with bilateral spastic cerebral palsy following treatment; HC, health children. N, number of participants; M, male, F, female; SD, standard deviation; Gesell, Gesell Developmental Schedules; GMFCS, Gross Motor Function Classification System; Gender was analyzed using chi-square test; Age, the scores of Gesell was analyzed using one-way analysis of variance. P < 0.05 indicated statistically significant differences*.

### Differences of Nodal Clustering Coefficient

**Figure 1:**
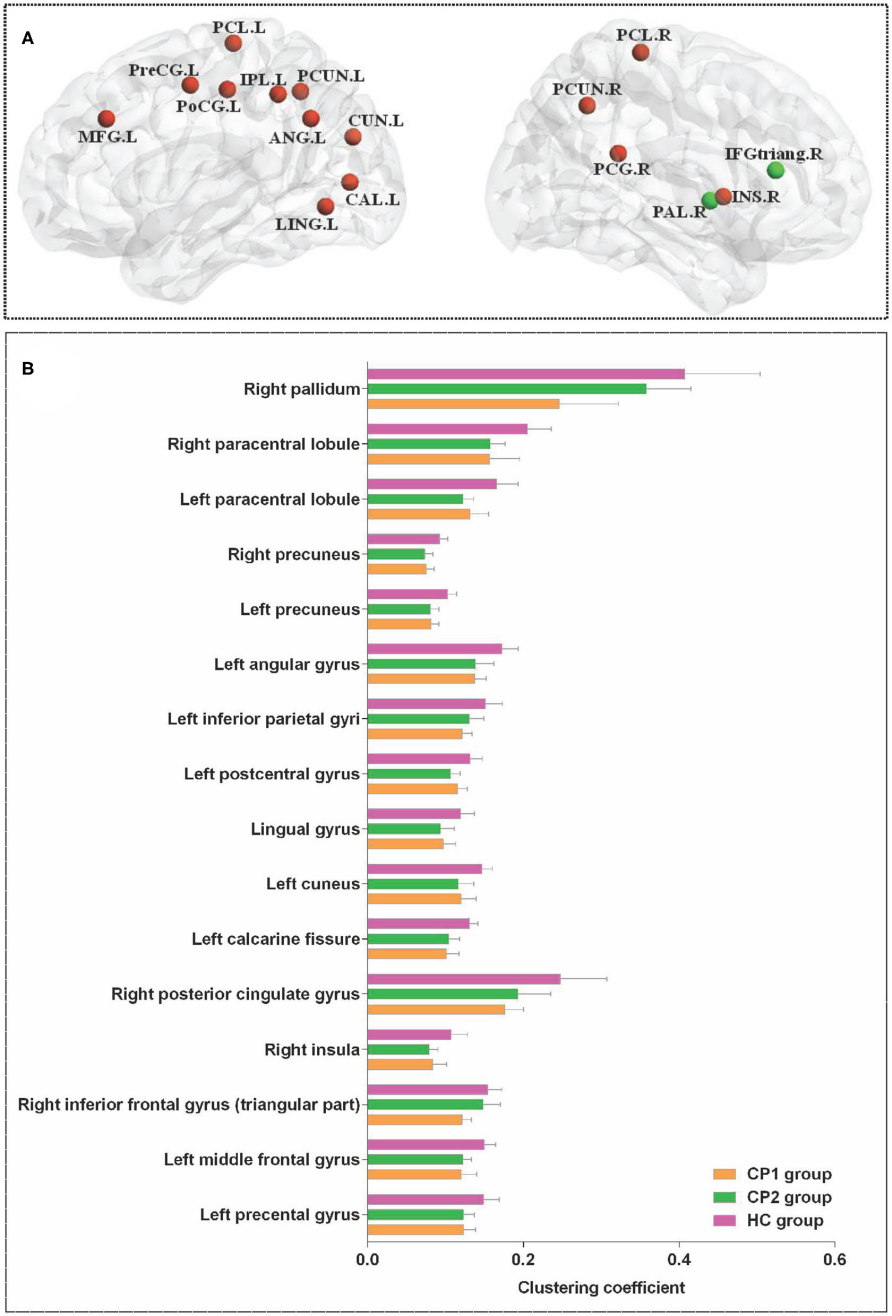
Brain regions with significant group effects in the nodal clustering coefficient among the three groups (ANOVA; FDR-corrected *P* < 0.05). CP1, children with bilateral spastic cerebral palsy before treatment; CP2,children with bilateral spastic cerebral palsy following treatment; HC, health children. **(A)** Brain regions with significant group differences; **(B)** statistical comparisons among the three groups. ANOVA: one-way analysis of variance. FDR: false discovery rate. FDR-corrected *P* < 0.05 indicated statistically significant differences among the three groups.

Figure 1 showed the brain regions with significant group differences in *C*_*i*_ (ANOVA; FDR-corrected *P* < 0.05). These regions were located in the left precental gyrus, middle frontal gyrus, calcarine fissure, cuneus, lingual gyrus, postcentral gyrus, inferior parietal gyri, angular gyrus, precuneus, paracentral lobule and the right inferior frontal gyrus (triangular part), insula, posterior cingulate gyrus, precuneus, paracentral lobule, pallidum (Table 2).

**Table 2:** Brain regions with significant group effects in the nodal clustering coefficient among the three groups (ANOVA; FDR-corrected *P*<0.05).

**Brain regions**	**Nodal clustering coefficient**			***F***	***P***
	**CP1**	**CP2**	**HC**		
Left precental gyrus	0.12 ± 0.016	0.12 ± 0.015	0.15 ± 0.020	11.07	0.00015
Left middle frontal gyrus	0.12 ± 0.020	0.12 ± 0.011	0.15 ± 0.015	16.04	0.000008
Right inferior frontal gyrus (triangular part)	0.12 ± 0.012	0.15 ± 0.023	0.15 ± 0.018	13.17	0.00004
Right insula	0.08 ± 0.018	0.08 ± 0.011	0.11 ± 0.021	10.60	0.0002
Right posterior cingulate gyrus	0.18 ± 0.024	0.19 ± 0.042	0.25 ± 0.060	9.76	0.000354
Left calcarine fissure	0.10 ± 0.016	0.10 ± 0.014	0.13 ± 0.011	20.23	0.000001
Left cuneus	0.12 ± 0.020	0.12 ± 0.020	0.15 ± 0.014	12.24	0.000071
Left lingual gyrus	0.10 ± 0.016	0.09 ± 0.018	0.12 ± 0.018	10.08	0.00029
Left postcentral gyrus	0.12 ± 0.012	0.11 ± 0.013	0.13 ± 0.016	12.00	0.000083
Left inferior parietal gyri	0.12 ± 0.013	0.13 ± 0.019	0.15 ± 0.022	10.58	0.00021
Left angular gyrus	0.14 ± 0.015	0.14 ± 0.024	0.17 ± 0.021	14.64	0.000017
Left precuneus	0.08 ± 0.010	0.08 ± 0.011	0.10 ± 0.012	18.83	0.000002
Right precuneus	0.07 ± 0.011	0.07 ± 0.011	0.09 ± 0.010	14.79	0.000016
Left paracentral lobule	0.13 ± 0.024	0.12 ± 0.014	0.17 ± 0.028	14.50	0.000018
Right paracentral lobule	0.16 ± 0.039	0.16 ± 0.020	0.21 ± 0.030	12.24	0.000071
Right pallidum	0.25 ± 0.076	0.36 ± 0.057	0.41 ± 0.096	15.86	0.000008

*CP1, children with bilateral spastic cerebral palsy before treatment; CP2, children with bilateral spastic cerebral palsy following treatment; HC, health children. ANOVA: one-way analysis of variance. FDR, false discovery rate. FDR-corrected P < 0.05 indicated statistically significant differences among the three groups*.

#### Nodal Clustering Coefficient in CP1 vs. HC

**Figure 2:**
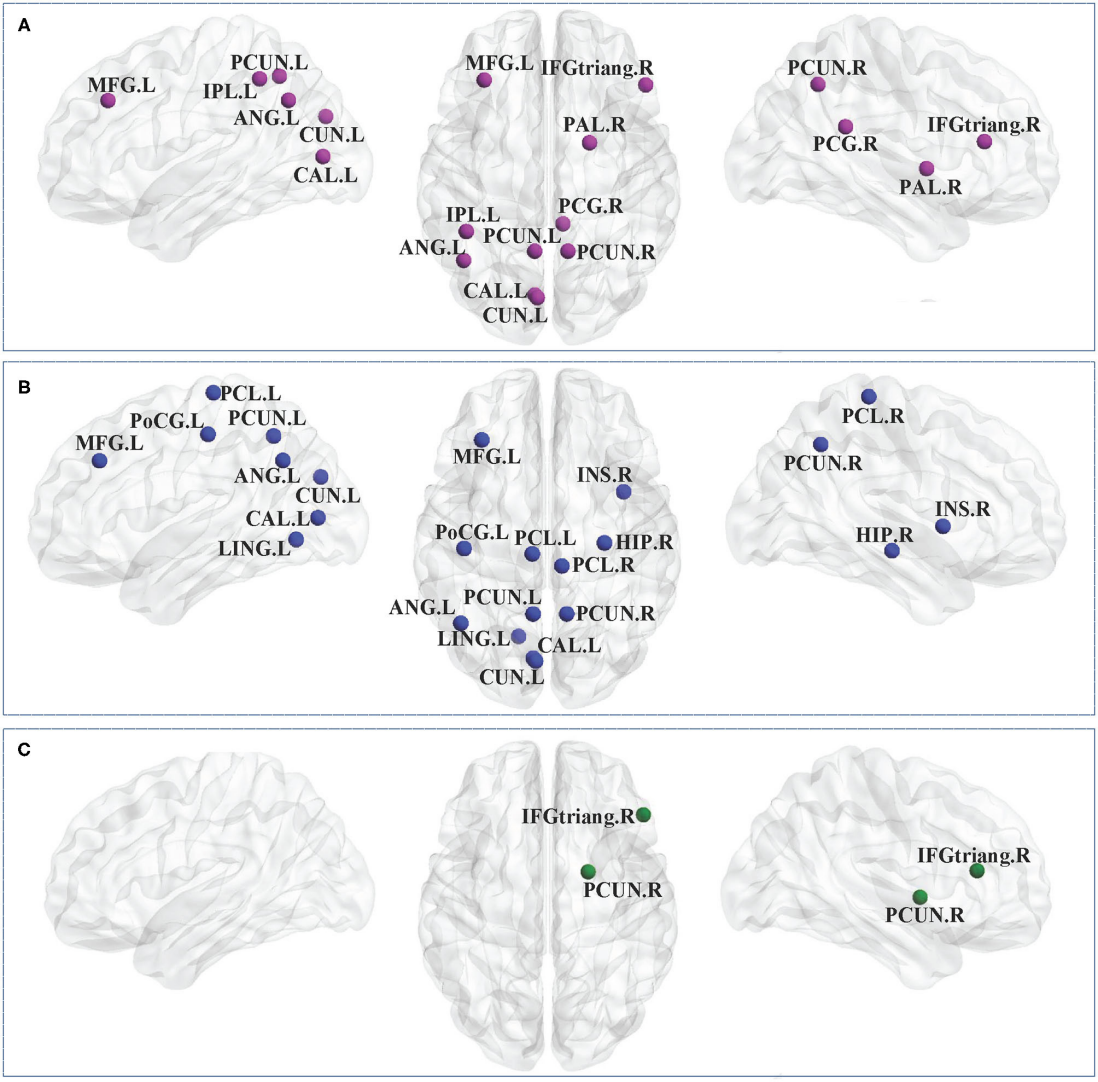
Brain regions with significant group effects in the nodal clustering coefficient between the CP1, CP2, and HC groups (*post hoc t*-test; FDR-corrected *P* < 0.05). CP1, children with bilateral spastic cerebral palsy before treatment; CP2, children with bilateral spastic cerebral palsy following treatment; HC, health children. **(A)** Statistical comparisons between CP1 and HC; **(B)** statistical comparisons between CP2 and HC; **(C)** statistical comparisons between CP1 and CP2. FDR, false discovery rate. FDR-corrected *P* < 0.05 indicated statistically significant differences between groups.

Between-group comparisons revealed that the CP1 group had a significantly decreased *C*_*i*_ in the left middle frontal gyrus, calcarine fissure, cuneus, inferior parietal gyrus, angular gyrus, precuneus and the right inferior frontal gyrus (triangular part), posterior cingulate gyrus, precuneus, pallidum when compared with the HC group (*post hoc t*-test; FDR-corrected *P* < 0.05; Figure 2 and Table 3).

**Table 3:** Brain regions with significant group effects in the nodal clustering coefficient between the CP1, CP2, and HC groups (*post hoc t*-test; FDR-corrected *P* < 0.05).

**Brain regions**		**Nodal clustering coefficient**		***t***	***P***
CP1 < HC	Left middle frontal gyrus	0.12 ± 0.020	0.15 ± 0.015	−4.50	0.00012
	Right inferior frontal gyrus (triangular part)	0.12 ± 0.012	0.15 ± 0.018	−5.62	0.000006
	Right posterior cingulate gyrus	0.18 ± 0.024	0.25 ± 0.060	−4.07	0.00037
	Left calcarine fissure	0.10 ± 0.016	0.13 ± 0.011	−5.73	0.000004
	Left cuneus	0.12 ± 0.020	0.15 ± 0.014	−4.26	0.00022
	Left inferior parietal gyri	0.12 ± 0.013	0.15 ± 0.022	−4.52	0.00011
	Left angular gyrus	0.14 ± 0.015	0.17 ± 0.021	−5.24	0.000016
	Left precuneus	0.08 ± 0.010	0.10 ± 0.012	−5.13	0.000022
	Right precuneus	0.07 ± 0.011	0.09 ± 0.010	−4.47	0.00013
	Right pallidum	0.25 ± 0.076	0.41 ± 0.096	−4.99	0.000032
CP2 < HC	Left middle frontal gyrus	0.12 ± 0.011	0.15 ± 0.015	−5.58	0.000006
	Right insula	0.08 ± 0.011	0.11 ± 0.021	−4.29	0.0002
	Right hippocampus	0.10 ± 0.016	0.13 ± 0.021	−4.18	0.00027
	Left calcarine fissure	0.10 ± 0.014	0.13 ± 0.011	−5.70	0.000005
	Left cuneus	0.12 ± 0.020	0.15 ± 0.014	−4.73	0.000064
	Left lingual gyrus	0.09 ± 0.018	0.12 ± 0.018	−4.02	0.00042
	Left postcentral gyrus	0.11 ± 0.013	0.13 ± 0.016	−4.61	0.000086
	Left angular gyrus	0.14 ± 0.024	0.17 ± 0.021	−4.14	0.00031
	Left precuneus	0.08 ± 0.011	0.10 ± 0.012	−5.21	0.000017
	Right precuneus	0.07 ± 0.011	0.09 ± 0.010	−4.89	0.000041
	Left paracentral lobule	0.12 ± 0.014	0.17 ± 0.028	−5.21	0.000017
	Right paracentral lobule	0.16 ± 0.020	0.21 ± 0.030	−5.03	0.000028
CP1 < CP2	Right inferior frontal gyrus (triangular part)	0.25 ± 0.076	0.36 ± 0.057	−3.96	0.00052
	Right pallidum	0.12 ± 0.012	0.15 ± 0.023	−4.39	0.00017

*CP1, children with bilateral spastic cerebral palsy before treatment; CP2, children with bilateral spastic cerebral palsy following treatment; HC, health children. FDR-corrected P < 0.05 indicated statistically significant differences between groups*.

#### Nodal Clustering Coefficient in CP2 vs. HC

As shown in Figure 2 and Table 3 (*post hoc t*-test; FDR-corrected *P* < 0.05), the CP2 group demonstrated decreased *C*_*i*_ in the left middle frontal gyrus, calcarine fissure, cuneus, lingual gyrus, postcentral gyrus, angular gyrus, precuneus and the right insula, hippocampus, precuneus, paracentral lobule when compared with the HC group.

#### Nodal Clustering Coefficient in CP2 vs. CP1

Compared with the CP1 group, the CP2 group exhibited increased *C*_*i*_ in the right inferior frontal gyrus and pallidum (*post hoc t*-test; FDR-corrected *P* < 0.05; Figure 2 and Table 3).

### Differences of Nodal Path Length

**Figure 3:**
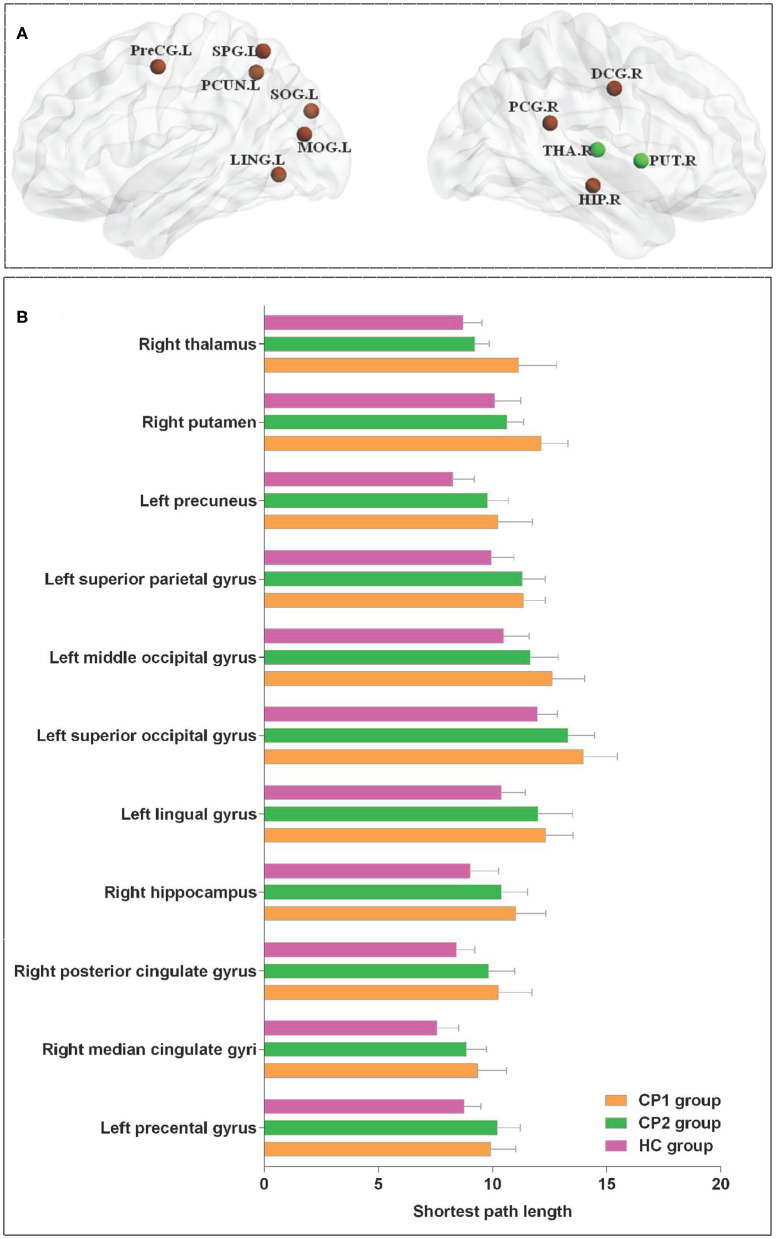
Brain regions with significant group effects in the nodal path length among the three groups (ANOVA; FDR-corrected *P* < 0.05). CP1, children with bilateral spastic cerebral palsy before treatment; CP2, children with bilateral spastic cerebral palsy following treatment; HC, health children. **(A)** Brain regions with significant group differences; **(B)** statistical comparisons among the three groups. ANOVA: one-way analysis of variance. FDR, false discovery rate. FDR-corrected *P* < 0.05 indicated statistically significant differences among the three groups.

Significant differences were detected among the three groups in the *L*_*i*_ of the left precental gyrus, lingual gyrus, superior occipital gyrus, middle occipital gyrus, superior parietal gyrus, precuneus and the right median cingulate gyrus, posterior cingulate gyrus, hippocampus, putamen, thalamus (ANOVA; FDR-corrected *P* < 0.05; Figure 3 and Table 4).

**Table 4:** Brain regions with significant group effects in the nodal path length among the three groups (ANOVA; FDR-corrected *P* < 0.05).

**Brain regions**	**Nodal path length**			***F***	***P***
	**CP1**	**CP2**	**HC**		
Left precental gyrus	9.90 ± 1.12	10.21 ± 1.02	8.76 ± 0.72	9.13	0.00054
Right median cingulate gyri	9.34 ± 1.27	8.84 ± 0.89	7.56 ± 0.95	11.29	0.00013
Right posterior cingulate gyrus	10.25 ± 1.47	9.81 ± 1.17	8.41 ± 0.81	9.75	0.00036
Right hippocampus	11.01 ± 1.33	10.37 ± 1.18	9.02 ± 1.24	9.68	0.00037
Left lingual gyrus	12.33 ± 1.20	11.98 ± 1.53	10.36 ± 1.05	9.94	0.00031
Left superior occipital gyrus	13.98 ± 1.49	13.29 ± 1.19	11.96 ± 0.89	10.60	0.0002
Left middle occipital gyrus	12.60 ± 1.43	11.64 ± 1.23	10.47 ± 1.14	10.30	0.00025
Left superior parietal gyrus	11.35 ± 0.97	11.29 ± 1.03	9.94 ± 0.99	9.43	0.00044
Left precuneus	10.23 ± 1.51	9.77 ± 0.91	8.25 ± 0.95	11.85	0.000091
Right putamen	12.12 ± 1.18	10.61 ± 0.75	10.09 ± 1.16	14.55	0.000018
Right thalamus	11.13 ± 1.68	9.21 ± 0.65	8.69 ± 0.84	18.08	0.000003

*CP1, children with bilateral spastic cerebral palsy before treatment; CP2, children with bilateral spastic cerebral palsy following treatment; HC, health children. ANOVA: one-way analysis of variance. FDR, false discovery rate. FDR-corrected P < 0.05 indicated statistically significant differences among the three groups*.

#### Nodal Path Length in CP1 vs. HC

**Figure 4:**
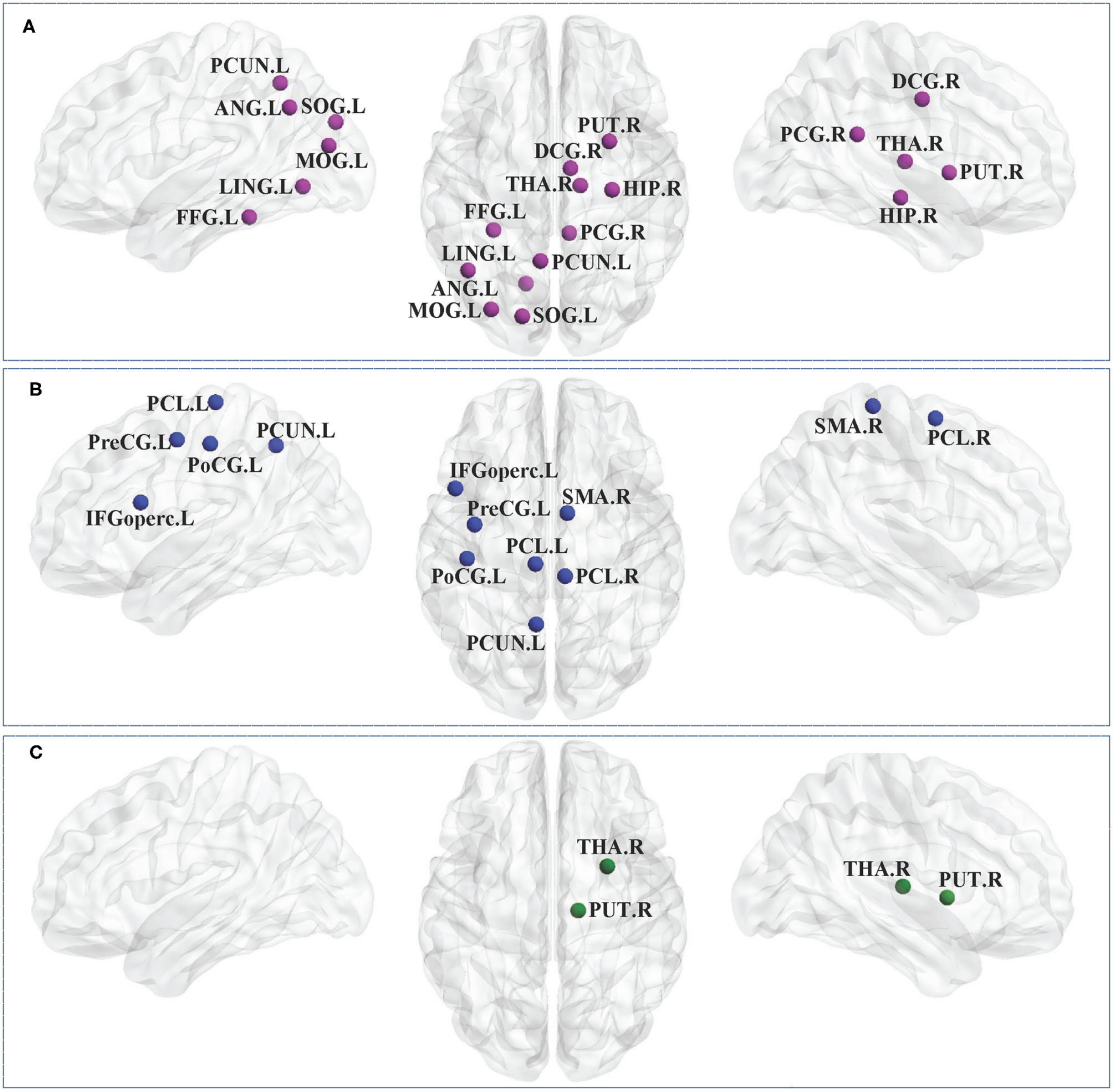
Brain regions with significant group effects in the nodal path length between the CP1, CP2, and HC groups (*post hoc t*-test; FDR-corrected *P* < 0.05). CP1, children with bilateral spastic cerebral palsy before treatment; CP2, children with bilateral spastic cerebral palsy following treatment; HC, health children. **(A)** Statistical comparisons between CP1 and HC; **(B)** statistical comparisons between CP2 and HC; **(C)** statistical comparisons between CP1 and CP2. FDR, false discovery rate. FDR-corrected *P* < 0.05 indicated statistically significant differences between groups.

The results showed that the CP1 group exhibited significantly increased *L*_*i*_ in the lingual gyrus, superior occipital gyrus, middle occipital gyrus, fusiform gyrus, angular gyrus, precuneus and the right median cingulated gyrus, posterior cingulate gyrus, hippocampus, putamen, thalamus when compared with the HC group (*post hoc t*-test; FDR-corrected *P* < 0.05; Figure 4 and Table 5).

**Table 5:** Brain regions with significant group effects in the nodal path length between the CP1, CP2, and HC groups (*post hoc t*-test; FDR-corrected *P* < 0.05).

**Brain regions**	**Nodal clustering coefficient**		***t***	***P***	
CP1>HC	Right median cingulate gyri	9.34 ± 1.27	7.56 ± 0.95	4.31	0.0002
	Right posterior cingulate gyrus	10.25 ± 1.47	8.41 ± 0.81	4.20	0.00026
	Right hippocampus	11.01 ± 1.33	9.02 ± 1.24	4.19	0.00027
	Left lingual gyrus	12.33 ± 1.20	10.36 ± 1.05	4.68	0.000071
	Left superior occipital gyrus	13.98 ± 1.49	11.96 ± 0.89	4.48	0.00012
	Left middle occipital gyrus	12.60 ± 1.43	10.47 ± 1.14	4.46	0.00013
	Left fusiform gyrus	13.91 ± 1.80	11.82 ± 0.94	3.94	0.00051
	Left angular gyrus	14.64 ± 1.89	11.92 ± 1.69	4.09	0.00035
	Left precuneus	10.23 ± 1.51	8.25 ± 0.95	4.26	0.00022
	Right putamen	12.12 ± 1.18	10.09 ± 1.16	4.70	0.000069
	Right thalamus	11.13 ± 1.68	8.69 ± 0.84	4.99	0.000031
CP2>HC	Left precental gyrus	10.21 ± 1.02	8.76 ± 0.72	4.43	0.00014
	Left inferior frontal gyrus (opercular part)	12.81 ± 1.17	11.24 ± 1.11	3.70	0.00098
	Right supplementary motor area	11.39 ± 0.74	10.12 ± 0.92	4.10	0.00034
	Left postcentral gyrus	10.26 ± 0.77	9.10 ± 0.78	4.04	0.0004
	Left precuneus	9.77 ± 0.91	8.25 ± 0.95	4.41	0.00015
	Left paracentral lobule	11.12 ± 0.84	9.65 ± 0.82	4.74	0.000061
	Right paracentral lobule	12.46 ± 1.10	10.70 ± 0.82	4.89	0.00004
CP1>CP2	Right putamen	12.12 ± 1.18	10.61 ± 0.75	4.05	0.00041
	Right thalamus	11.13 ± 1.68	9.21 ± 0.65	3.99	0.00048

*CP1, children with bilateral spastic cerebral palsy before treatment; CP2, children with bilateral spastic cerebral palsy following treatment; HC, health children. FDR-corrected P < 0.05 indicated statistically significant differences between groups*.

#### Nodal Path Length in CP2 vs. HC

Figure 4 and Table 5 showed that the CP2 group had a significantly increased *L*_*i*_ in the left precental gyrus, inferior frontal gyrus (opercular part), postcentral gyrus, precuneus, paracentral lobule and the right supplementary motor area, paracentral lobule when compared with the HC group (*post hoc t*-test; FDR-corrected *P* < 0.05).

#### Nodal Path Length in CP2 vs. CP1

*Post hoc t*-test revealed that the CP2 group had decreased *L*_*i*_ in the right putamen and thalamus when compared with the CP1 group (*post hoc t*-test; FDR-corrected *P* < 0.05; Figure 4 and Table 5).

## Discussion

Using DTI data and the graph theoretical analysis, we investigated the underlying neural changes following the treatment of rTMS in children with BSCP. The results showed significant differences of node-level metrics in various brain regions, which indicated a disruption in structural brain connectivity in BSCP. Children with BSCP showed significant differences of *C*_*i*_ and *L*_*i*_ in widespread brain areas including the cortical and subcortical regions when compared with HC. In addition, increased *C*_*i*_ of the right inferior frontal gyrus, pallidum and decreased *L*_*i*_ of the right putamen, thalamus were found in the BSCP children following the treatment of rTMS when compared with those of patients before treatment. These findings extended our understanding of the neurophysiologic mechanisms involved in BSCP and the effect of rTMS on changes in structural brain connectivity of BSCP from a network perspective.

Previous neuroimaging studies found that CP children were often associated with diffuse periventricular leukomalacia, which was a high risk factor for CP (31–33). In addition, it had been shown that structural alterations in CP children were not confined to a periventricular leukomalacia or sensorimotor-related brain regions, but were rather widespread across the whole brain of children (34–37). Consistent with the results of previous studies in children with CP (38–40), our results of ANOVA showed that BSCP children had topological alterations of extensive areas in the structural brain networks, which were primarily located in the prefrontal, parietal and occipital cortex, as well as some subcortical regions. The decreased *C*_*i*_ and increased *L*_*i*_ suggested that BSCP children lower structural connections between these regions and other brain regions than HC across the whole brain. These impaired topological architecture implied that the brain network of BSCP children had inefficient information exchange than HC.

Our results also confirmed the clinical therapeutic effect of rTMS on motor and cognition recovery in children with BSCP, which were consistent with the findings of previous studies. It had been identified that the treatment of rTMS could modulate the neuroplasticity below the magnetic coil, as well as the remote cortical and subcortical regions through structural connectivity (41). Children with BSCP in this study showed rTMS-induced changes of the topological characteristics of the structural brain networks. Increased *C*_*i*_ of the right inferior frontal gyrus, pallidum were found in BSCP children following the treatment of rTMS, as well as decreased *L*_*i*_ of the right putamen, thalamus. These results suggested that rTMS could modulate structural connectivity within the prefrontal basal ganglia circuits in the brain of BSCP.

Functional MRI study found that children with spastic diplegic CP had decreased regional homogeneity (ReHo) in the bilateral inferior frontal gyrus when compared with HC (42). Abnormal neural activity within the inferior frontal gyrus during the motor planning stage was also identified in children with CP, which was considered to be associated with tasks that required sustained vigilance (43, 44). The inferior frontal gyrus was also found to be involved in the process of executive planning and was necessary for matching the prescribed target forces (45). Based on these findings, it was possible that the impaired nodal parameters in the inferior frontal gyrus might indicate that children with BSCP had greater difficulty in sustaining their attention on the motor task demands.

Moreover, previous study with the network-based statistic (NBS) analysis demonstrated that fractional anisotropy (FA) with basal ganglia (mainly putamen and pallidum) and thalamus were significantly reduced in CP children (40). Gray matter lesions were also found in the basal ganglia of CP children, mainly located in bilateral putamen (46). The damaged posterior thalamic radiation connecting the thalamus to the posterior parietal and occipital cortex, was found to be associated with motor dysfunction in children with spastic CP (47). The motor network of CP was composed of the prefrontal, parietal regions, basal ganglia and thalamus (37), which were the most commonly affected subcortical structures in CP (40). The thalamus is the information integration center in the brain and is in the cortical-striatum-thalamus pathway (48). Therefore, impaired nodal metrics of basal ganglia and thalamus might be indirectly modulated by the stimulation of the cortical areas (49). The treatment of rTMS could modulate brain structural networks in children with BSCP and the modulated brain regions were associated with the improved motor and cognitive symptoms.

Several limitations should be noted in the present study. First, this was a small sample study and further studies with larger samples of children with CP were needed to advance the ability to generalize treatment outcomes of rTMS based on the method of MRI. Secondly, previous neuroimaging studies had identified aberrant functional connectivity in the brain of children with CP, therefore, future studies should employ structural-functional consistency analyses to fully elucidate the pathogenesis of CP. Thirdly, it was difficult to estimate the potential contribution of medication in relation to our results.

## Conclusion

In conclusion, the present study revealed impaired topological organization in the prefrontal, parietal, occipital cortex and some subcortical regions in BSCP children. On the other hand, the results showed significant improvement of motor and cognitive function and changes of nodal parameters in the prefontal-striatum-thalamus of BSCP children in response to the rTMS treatment. All these findings suggested that rTMS might improve clinical symptoms by modulating structural connectivity connecting to the prefontal-striatum-thalamus pathway for patients with BSCP.

## Data Availability Statement

The raw data supporting the conclusions of this article will be made available by the authors, without undue reservation.

## Ethics Statement

The studies involving human participants were reviewed and approved by the Ethics Committee of the Children's Hospital of Nanjing Medical University. Written informed consent to participate in this study was provided by the participants' legal guardian/next of kin.

## Author Contributions

LZ, HX, JT, and XZ designed the experiments. MZ, YW, YL, WZ, and SZ contributed to clinical data collection and assessment. LZ and HX analyzed the results. LZ, HX, WZ, and SZ wrote the manuscript. JT and XZ approved the final manuscript. All authors contributed to the article and approved the submitted version.

## Conflict of Interest

The authors declare that the research was conducted in the absence of any commercial or financial relationships that could be construed as a potential conflict of interest.
